# The management of talar osteochondral lesions - Current concepts

**DOI:** 10.1016/j.jajs.2021.04.002

**Published:** 2021

**Authors:** Tian Lan, Helen S. McCarthy, Charlotte H. Hulme, Karina T. Wright, Nilesh Makwana

**Affiliations:** a Spinal Studies & Cartilage Research Group, Robert Jones and Agnes Hunt Orthopaedic Hospital, NHS Trust, Oswestry, UK; b Robert Jones and Agnes Hunt Orthopaedic Hospital, NHS Trust, Oswestry, UK; c School of Pharmacy and Bioengineering, Keele University, UK

**Keywords:** Ankle, Talus, Osteochondral lesion, Osteochondral defect, Treatment

## Abstract

Osteochondral lesions of the talus (OLTs) are a common complication following trauma, involving both the articular cartilage and the underlying subchondral bone, with variable aetiologies and often presenting with non-specific symptoms. Diagnosis of OLTs requires a combination of clinical assessment and imaging and despite many different treatment options, there is no generalised consensus regarding which option is the most effective. Left untreated, OLTs risk progressing to osteoarthritis. Acute non-displaced OLTs can be treated non-operatively. However, OLTs refractory to non-surgical care for three to six months may be suitable for surgical care. In these cases, conservative treatments are often unsuccessful, particularly for larger and more severe defects and so the majority require surgical intervention. Although bone marrow stimulation techniques remain the “gold standard” for lesions <150 mm^2^, there still requires a need for better long term clinical data and cost-benefit analyses compared with other treatment options. Biological attempts at either regenerating or replacing the articular cartilage are however demonstrating some promising results, but each with their own advantages and disadvantages. In this review, we summarise the clinical management of OLTs and present the current concepts of different treatment regimes.

## 1 Introduction

Osteochondral lesions of the talus (OLTs), also known as talar osteochondral defects, include many varieties of pathologies such as osteochondritis dissecans, transchondral fractures and osteochondral fractures. In general, OLTs refer to any defect of the ankle articular cartilage and underlying subchondral bone.^1^ OLTs are mainly associated with trauma, with the majority of defects occurring in young people aged between 20 and 40 years, following ankle sprains or fractures.^2^ They can be diagnosed by plain radiography, computerized tomography (CT), magnetic resonance imaging (MRI) and arthroscopy; OLT classifications among these diagnostic techniques also vary.^3^ Patients with symptomatic OLTs will normally suffer from prolonged pain, swelling, locking and catching of the ankle joint. Acute non-displaced OLTs can be treated non-surgically with successful results in up to 50% of cases.^4^^,^^5^ Due to the low healing capacity of articular cartilage, conservative treatments such as immobilisation, rest and restriction of activities do not have a good performance in ankle restoration in late stage symptomatic OLT patients.^6^ Untreated OLTs may contribute to the development of early-stage osteoarthritis (OA) and lead to the severe disability.^7^^,^^8^ Bone marrow stimulation is still a gold standard among surgical interventions for lesions <150mm^2^, but it cannot promise good long-term results.^1^^,^^9^ Newer, novel cell-based therapies are developing rapidly, aiming for safe, cost-effective and improved long-term results.

## 2 Aetiology

The aetiology of OLTs is still controversial but are more common in young and active patients (20–40 years old) following an ankle sprain or trauma.^2^^,^^10^ It is widely accepted that OLTs are caused by both traumatic and nontraumatic events with up to 50% of ankle sprains and over 70% of ankle fracture cases leading to the development of osteochondral lesions.^7^^,^^11^ A study by Tol et al. (2000),^12^ demonstrated 93% of patients with lateral lesions had a history of trauma, compared to only 62% with medial lesions. According to Berndt and Harty,^13^ the mechanism of injury in lateral lesions, is associated with ankle joint dorsiflexion and inversion, while in medial lesions it is associated with ankle plantarflexion and inversion. OLTs without trauma history could possibly be caused by repetitive microtrauma, ischemia, subsequent avascular necrosis, genetic predisposition and endocrine or metabolic factors.^14^

**Fig. 1:**
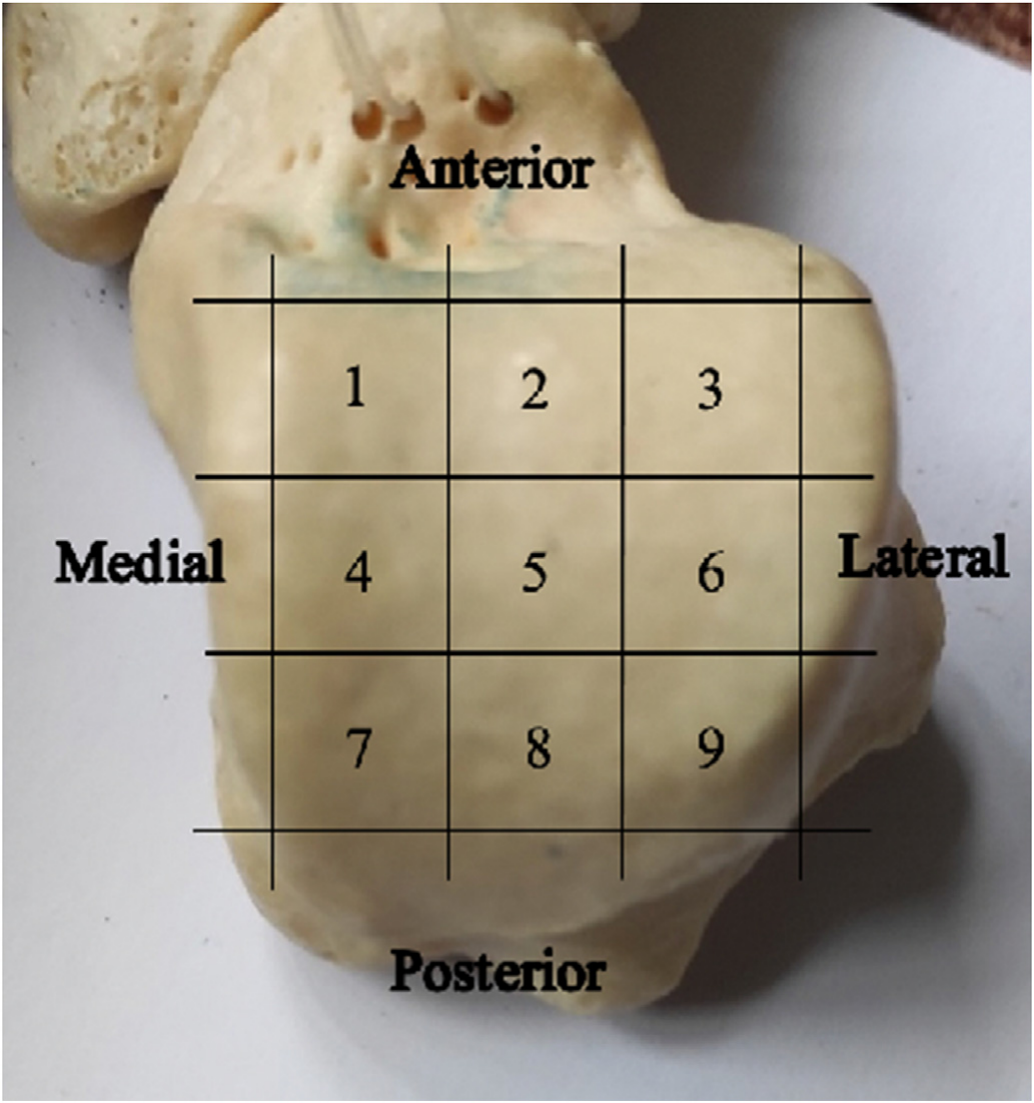
**Anatomical 9 zone grid system of the talar dome.**^15^ The talar dome is divided into 9 equal zones, with zones 1, 2 and 3 located anteriorly and zones 3, 6 and 9 located laterally.

Depending on their anatomical location, OLTs are divided into different types. Posteromedial and anterolateral OLTs were traditionally thought to be the most common OLTs.^13^ Raikin et al. (2007) examined 424 patients by MRI based on a 9-zone anatomic grid system (Fig. 1), and found that the majority of lesions (53%) were located in zone 4 (centromedial), and that the second most common lesion location was zone 6 (centrolateral).^15^ Another study, however, demonstrated the opposite with regards symptomatic and operatively treated lesions, with the centrolateral region being twice as common as the centromedial region of the talar dome.^16^ Despite this, these studies demonstrate that the centrolateral and centromedial areas of the talar dome are the most common locations for OLTs. In addition, it has been shown in several studies that medial lesions are generally larger and deeper while lateral lesions are smaller and shallower.^13^^,^^15^^,^^16^ However, lateral lesions tend to be more symptomatic than medial lesions, possibly due to the higher baseline contact pressure found in the centrolateral area compared to the centromedial area of the ankle joint.^16^

It has been demonstrated that the posteromedial and anterolateral regions of the talar dome have the thickest articular cartilage, as evidenced by quantitative cartilage thickness measurements of 12 cadaveric ankle joints by high resolution stereophotography.^17^ The authors of this study have related this finding to an adaptive response of the tissue in this region to the greatest mechanical stress, and claimed that these results are consistent with the most common sites of OLTs.^17^ However, other studies suggest that the posterolateral region of the talar dome has the thickest articular cartilage and that the anterolateral region has the thinnest articular cartilage, as assessed by confocal microscopy and MR imaging with 3D modelling respectively.^18^^,^^19^ Further, Sugimoto et al. (2005) concluded that articular cartilage in the medial corner of the talar dome showed the greatest thickness, while the lateral gutter of the talar dome showed the least thickness, as assessed radiographically.^20^ Taken together, although there is no complete agreement on the thickness of cartilage in specific regions, the cartilage thickness has been shown to vary in all studies across the talar dome, and its relationship with the mechanism of OLT development requires further investigation.

Compared to the knee, the ankle is anatomically different: it has a smaller articular contact area than the knee, and over 60% of the talar dome is covered by hyaline cartilage. Ankle cartilage has a higher dynamic stiffness due to the higher proteoglycan content, which contributes to stability when weightbearing or loading.^21^ The average cartilage thickness of the ankle is almost two folds thinner than the knee, however a study showed that the ankle has a relatively higher proportion of the superficial zone of cartilage, which indicates that ankle has stronger protection than the knee when weightbearing.^22^ In addition, ankle articular cartilage has undetectable levels of matrix metalloproteinase-8 (MMP-8) compared to knee articular cartilage, reducing endogenous degradation of extracellular matrix proteins.^23^ From a biomechanical point of view, the main movements of the ankle joint are rolling and rotating, while the knee experiences higher shear forces from its rolling, sliding and rotating movements.^24^ Although the development of ankle OA is considered to be associated with untreated OLTs,^7^^,^^8^ the incidence of OA in the ankle is relatively low; around 15% of adults are affected by OA worldwide, however, only 1% of this is ankle OA.^25^ In a 21-year follow up study, Bauer et al. (1987) found that only two out of 30 patients’ OLTs had progressed on to develop OA.^26^ According to Günther et al. (1998), 67% of knee OA cases were primary origin,^27^ while Valderrabano et al. (2008) reported that primary ankle OA only accounts for 9% of cases, whilst 78% of OA cases were posttraumatic.^25^ In conclusion, the ankle develops less OA than the knee,^28^ with ankle OA developing mainly post-traumatically and knee OA developing idiopathically.^24^

## 3 Diagnosis and evaluation

Pain, swelling and stiffness are the most common complaints of presenting patients with OLTs, occurring particularly with high levels of activity such as sport, but rarely present at rest.^29^ Occasionally, there may also be mechanical symptoms present such as locking and catching.^3^ Patients will often relate the pain experienced to a history of either a single trauma or recurrent sprains. Often the initial OLT is misdiagnosed as an ankle sprain.^30^ Chronic OLTs present with non-specific symptoms, in these cases, physical examination may show an effusion, decreased range of motion, general tenderness, and pain with inversion or dorsiflexion.^29^ In general, patients often describe a deep-seated pain with more chronic OLTs compared with a more superficial pain with synovitis.^31^ Of course, the absence of physical signs and symptoms on examination does not exclude the presence of OLTs, highlighting the importance of further clinical imaging investigations being required to confirm OLT diagnosis.^31^

Imaging modalities most commonly utilised in the clinic for the identification and diagnosis of OLTs include plain radiography, MRI and CT, each with their own benefits, drawbacks and classification systems (Table 1). Initially, plain radiographs are often used upon first presentation usually taken in anterior-posterior (AP) mortise view and lateral weight-bearing views of the ankle. In the acute injury a trauma series is often taken. Radiographs have relatively low sensitivity and are often unclear, missing over 40% of cases.^31^^,^^32^ The Berndt and Harty radiography score is a 4-stage classification system that assesses the initial subchondral compression (stage I) and osteochondral fragments being either partially detached (stage II), completely detached (stage III) or displaced (stage IV; Table 1).^13^

**Table 1:** Overview of the classification systems used across the different assessment modalities used in the diagnosis of OLTs. OC = osteochondral fragment; SCB = subchondral bone.

X-Ray	MRI	CT	Arthroscopy	
Berndt and Harty^13^	Hepple^34^	Ferkel^36^	ICRS^37^	Cheng-Ferkel^38^
I – subchondral compression	1 - articular damage only	I - cystic lesion, intact overlying articular cartilage	1 – superficial zone softening or fissure	A – smooth and intact cartilage, but soft
II – partially detached OC fragments	2a – Articular cartilage damage with subchondral fracture (- odema)	IIA – cystic lesion with articular surface communication	2 – lesions extend <50% depth	B – rough articular surface
III – fully detached OC fragments	2b – Articular cartilage damage with subchondral fracture (+odema)	IIB – overlying non-displaced OC fragment	3 – lesions extend >50% depth but not into SCB	C – fissures and fibrillations present
IV – displaced OC fragments	3 – detached non-displaced OC fragment	III – non-displaced OC fragment with lucency	4 – lesion extends into the SCB	D – cartilage flap or exposed SCB
	4 – detached and displaced OC fragment	IV – displaced OC fragment		E − loose, non-displaced fragments
	5 – subchondral cysts			F – displaced fragment

MRI is more able to clearly visualise soft tissues such as articular cartilage, synovium and tendons, making it advantageous in detecting and characterising OLTs.^31^ It is often the imaging modality of choice following inconclusive plain radiographs or continuation of symptoms. However, MRI is less able to assess bony changes and can make it difficult to fully assess the true status of the subchondral bone and the exact lesion dimensions.^33^ OLTs on MRI are classified according to the 5-stage Hepple MRI classification system,^34^ where stage 1 is articular cartilage damage only, stage 2 is cartilage damage with subchondral fracture (this is further categorised as either acute (2a) or chronic (2b) dependent upon the presence of oedema), stages 3 and 4 include detached non-displaced and displaced osteochondral fragments, respectively and stage 5 is the presence of subchondral cysts (Table 1).

CT on the other hand provides a far superior assessment of the subchondral bone compared to MRI and can therefore more accurately predict the depth of the OLT unless it is an early lesion, which is characterised by changes in the articular cartilage.^35^ Performing a CT scan can provide the surgeon with the finer details required for accurate surgical planning.^35^ OLTs on CT are classified according to the Ferkel system where stage I is the presence of a cystic lesion but with intact overlying articular cartilage,^36^ stage IIA is where the cystic lesion communicates with the articular surface and stage IIB includes an overlying non-displaced osteochondral fragment, stage III is a non-displaced fragment with lucency beneath it and stage IV is a displaced fragment (Table 1).

Arthroscopy is also an effective method for visualising and investigating OLTs. The ability to assess the entire joint in addition to probing the lesion can provide the most comprehensive evaluation in deciding the most appropriate treatment regime. Arthroscopic evaluation of OLTs uses the International Cartilage Repair System (ICRS) grading,^37^ which is a standardised grading system. Grade 1 lesions involve the superficial zone with softening or fissure, Grade 2 lesions extend less than 50% of the depth, Grade 3 greater than 50% in the depth but not to the subchondral bone as in Grade 4 (Table 1). Cheng-Ferkel grading is another arthroscopic grading system used in OLT evaluation, Grade A lesions present smooth and intact cartilage with a soft or ballottable arthroscopic appearance, Grade B lesions show a rough surface, Grade C lesions appear fibrillations or fissures, Grade D lesions have a flap present or bone exposed, Grade E lesions are loose and normally show non-displaced fragments and displaced fragments present in Grade F lesions (Table 1).^38^

## 4 Treatment and outcomes

**Fig. 2:**
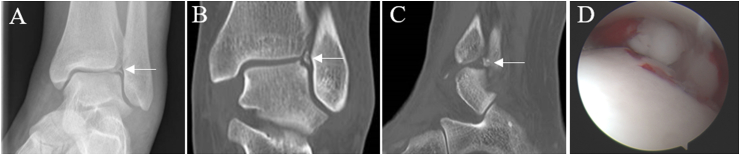
**22-year-old female case presenting with an acute injury, with a history of a fall from a horse.** X-ray (A) and CT (B–C) imaging demonstrated lateral talar dome OLTs (arrow). OLT fragment was excised arthroscopically (D).

The goal of any treatment strategy of course is to diminish debilitating symptoms such as pain and swelling and to improve function. The effectiveness of each strategy, however, is variable and often the strategy adopted is based not only on the type and size of the defect but also preferences of the treating clinician. In acute non-displaced OLTs injuries conservative methods of treatment are usually considered with the intention of reducing the load on the damaged cartilage to resolve bone oedema or encourage healing of any detached fragments.^39^ Methods include rest and/or restriction of activities with or without the use of non-steroidal anti-inflammatories (NSAIDs), short-term immobilisation in a cast or boot for approximately 6 weeks, followed by physiotherapy and progressive weight-bearing with a slow return to previous activities.^1^^,^^29^ Such conservative techniques are effective in only ∼50% of cases.^39^ Chronic symptomatic OLTs, acute fragmented OLTs and those refractory to conservative management should be considered for surgical intervention.^1^ Acute displaced, symptomatic OLTs can be treated by open or arthroscopic methods, whereas large OLTs are usually fixed using headless cannulated screws or absorbable pins. Access to the ankle is restricted and this may require either a medial or lateral malleolar osteotomy for access. Small or fragmented lesions can simply be excised (Fig. 2). Symptomatic OLTs that are chronic or refractory to non-surgical measures should be considered for surgical intervention. Surgical interventions to treat OLTs are most commonly performed as arthroscopic interventions. The majority of procedures treat the OLTs through debridement of any damaged hyaline cartilage and underlying subchondral bone. The ‘gold standard’ treatment for lesions <150 mm^2^ remains microfracture,^1^^,^^9^ which involves creating small holes in the subchondral bone to create a new blood supply, also resulting in innate mesenchymal stem or stromal cells (MSCs) entering the defect from the subchondral blood/marrow and stimulating healing of the cartilage. Microfracture benefits patients who require quick return to function, as this procedure requires early mobilisation of the joint through its’ associated recommended rehabilitation programme.^40^ Optimal clinical improvements of microfracture (based on Visual Analogue Scale (VAS) and American Orthopaedic Foot and Ankle Society (AOFAS) score) have been demonstrated at 24 months post-operatively.^41^ Recently published findings from both Kim et al. (2019) and Choi et al. (2020) indicate that microfracture results in mid-term improvements.^41^^,^^42^ When assessing 70 patients, 85% of patients showed some improvement 6 months after their microfracture, for whom their symptoms did not worsen from baseline at 3 years post-operatively.^41^ Further, in a study of 156 patients (165 ankles) improvement or maintenance of pain and functional scores (AOFAS; VAS; Short Form-36) compared with baseline scores could be demonstrated up to six years post-operatively.^42^ There does appear to be an inverse relationship between the size of the lesion and the outcome following microfracture. The critical size of the defect appears to be those that are less than 150 mm^2^.^41^^,^^43^ Interestingly, a systematic review of 70 patients indicated that microfracture might also be effective as a treatment for non-primary OLTs.^44^

In patients where lesions are observed within the subchondral bone but the hyaline cartilage is intact, retrograde drilling can be offered with the aim of treating the subchondral lesion whilst preserving overlying cartilage.^45^ In the retrograde drilling procedure, articular cartilage is selected for entry into the talus and then a guidewire is directed into the lesion in a retrograde direction under x-ray control. This can also be under computer navigation to place the guide wire and direct the location of drilling.^45^

Other interventions used to treat OLTs include those in which osteochondral transplants are inserted into small defect. These can be in the form of Osteochondral Autograft Transfers or Osteochondral Allograft Transplantations (OATs). In either of these procedures, non-weight bearing cartilage and subchondral bone is harvested as a cylindrical plug which is matched in size and area to the problem defect. To treat larger defects, mosaicplasty can be performed in which multiple smaller osteochondral (OC) plugs are taken and inserted into the defect(s).^46^ For defects that are less than 1 cm^2^, OATs are more commonly used in which the OC plugs are harvested from the patient themselves. Where defects are larger than 2 cm^2^, OATs are carried out using arthrotomy. This relies on the harvest of OC plugs from cadaveric donors and subsequent sterilisation of the tissue prior to being transplanted into the patient’s defect site. OATs have long been turned to as a surgical option for the treatment of OLTs, with these surgeries having been performed widely since 1992.^47^ A number of systematic reviews have recently been published which aimed to determine the clinical outcomes following OATs in the ankle.^48^ Pereira et al. (2021), found that in 12 studies, yielding 191 patients, there were no short-term complications following fresh OATs and that the graft survival rate was 86.6% (assessed by AOFAS and VAS score).^48^ Autologous osteochondral transplants were also concluded to have good clinical outcomes (AOFAS, MRI and radiographs) at a mean of 62.8 months follow-up, although donor site morbidity was demonstrated in 18 of the 500 study participants.^49^

More recently, surgeons have been investigating the potential of another allogeneic tissue donor source which uses particulated juvenile articular cartilage (PJAC).^50^ These juvenile grafts are taken from donors typically younger than 13 years old. The only commercial graft available for this procedure at present, is DeNovo NT Natural Tissue Graft (Zimmer, Warsaw, IN, USA), marketed as a pre-packaged allograft consisting of immature chondrocytes in their innate extracellular matrix. These grafts can be used to treat focal defects up to 2 cm^2^ with fibrin used to adhere the allograft in place. Similar to osteochondral allograft transfer, this procedure benefits from removing the possibility of donor site morbidity that comes from an autologous tissue transfer procedure.^51^ PJAC treatment has been reported to produce articular cartilage, which is more akin to native tissue.^50^ Aldawsari et al. (2021), found that of the published 10 studies of PJAC radiological and clinical outcomes included in their systematic review, PJAC demonstrated promising functional outcomes, with MRIs demonstrating some filling of the OLTs.^52^ However, there was some disparity in cartilage repair between studies and a consistent lack of repair in the subchondral bone and subchondral lamina.^52^ Furthermore, a comparative study found PJAC had no advantage over microfracture based on patient reported outcomes.^53^

Cell-based surgical interventions are regenerative medicine methods used to biologically regenerate the cartilage rather than replace it. Techniques include autologous chondrocyte implantation (ACI), autologous matrix-induced chondrogenesis (AMIC), platelet-rich plasma (PRP) and bone-marrow aspirate concentrate (BMAC). These treatment modalities are often used following a failed microfracture.^54^ ACI is a long-established 2-stage procedure initially developed for chondral/osteochondral lesions in the knee,^55^ that, in the ankle, involves harvesting macroscopically normal hyaline cartilage either from the anterior talus or detached osteochondral fragments.^56^^,^^57^ Previously, harvests for ankle ACI have been obtained from the knee, with limited donor-site morbidity.^58^^,^^59^ Chondrocytes are enzymatically extracted from the cartilage and culture-expanded *in* *vitro* prior to being re-implanted into the defect some 3–4 weeks later, beneath either a periosteal patch or a commercially available type I/III collagen patch.^60^ Alternatively, extracted chondrocytes can be cultured directly on the collagen patch, a procedure known as matrix-assisted ACI, or MACI. Pagliazzi et al. (2018) demonstrated a significant improvement in the patient AOFAS score at 7 years (87.2 ± 14.5 months) following the ACI.^61^ Despite the recent technical appraisal by the National Institute for Clinical Excellence (NICE) approving the use of ACI in the knee,^62^ the same cannot be said for the ankle. Current guidelines state that ACI will not be routinely commissioned due to lack of evidence of the clinical effectiveness of this therapy in the ankle, so it remains a clinical trial/efficacy study line of treatment only at present (within the UK at least).

To eradicate the need for a 2-stage procedure, the AMIC technique offers a promising, cost-effective alternative that was originally developed for the treatment of defects in the knee.^63^ The technique consists of performing a microfracture followed by securing a commercially available type I/III collagen patch over the defect with the aim of not only trapping any MSCs entering the defect area through the bleeding process, but also providing a scaffold on which the cells may adhere and proliferate.^64^ It has been suggested that transforming growth factor beta (TGF-beta) present in the fibrin glue used to adhere the patch may enhance MSC chondrogenic differentiation.^65^ However, a recent systematic review of AMIC in the talus concluded that more clinical studies are required to validate the efficacy and safety of this technique.^66^ Complete defect filling was demonstrated in 88% of patients (n = 33) treated with AMIC when assessed by MRI, along with reduction in pain and improvement in function at 4.7 (mean; range, 2.3–8.0) years post-operatively.^67^ A systematic review of 13 papers, further confirmed the efficacy of AMIC,^66^ as did another of 15 studies with outcomes up to five years post-operatively.^68^

There has been increasing interest in recent years with regards to a biological therapy for OLTs utilising PRP, either autologous or allogenic, which can either be administered directly to the lesion or via intra-articular injection.^69^^,^^70^ PRP is rich in various growth factors and cytokines such as TGF-beta, platelet-derived growth factor (PDGF), insulin-like growth factor (IGF) I and II, fibroblast growth factor (FGF) and vascular endothelial growth factor (VEGF) which are believed to enhance the natural healing response by promoting MSC differentiation and cartilage formation.^71^, ^72^, ^73^ The use of PRP to treat OLTs has also been systematically reviewed and was concluded to result in significantly reduced pain and improved function in comparison with microfracture alone.^73^ Despite being a promising treatment option, to date, there is limited clinical evidence for the use of PRP and an absence of a standardised preparation technique prior to application.^74^

BMAC is a type of biological adjuvant used for one-step cartilage repair of OLTs, in the most recent decade.^75^ BMAC consists of different types of stem cells and progenitor cells such as MSCs and haematopoietic progenitor cells (HPCs), which have the potential to repair injured hyaline cartilage. BMAC also contains an abundance of growth factors and cytokines which may contribute to the cartilage regeneration process.^76^ In BMAC therapy, bone marrow aspirate is collected from the patient’s iliac crest, the white cells in the marrow including MSCs, HPCs and all other immune cell fractions are concentrated with the aid of commercial kits, and injected directly into the patient’s OLT, combined with a biological scaffold, such as a hyaluronic acid and fibrin gel.^77^ Unlike the two-stage procedure of ACI, BMAC therapy can be achieved during a single surgical operation, and does not require *in vitro* cell culture, which significantly lowers the financial burden (as BMAC is not considered an advanced therapy medicinal product by regulators), and avoids the heterogeneity caused by inconsistent cell culture protocols between laboratories. However, BMAC therapy still lacks standardisation; there is no defined protocol for bone marrow aspirate collection and concentration, and the scaffolds used in the treatment vary.

BMAC has been demonstrated to have good long-term results in the treatment of both OLTs and OA.^77^, ^78^, ^79^ In one of these studies patients receiving BMAC therapy demonstrated significantly increased AOFAS scores, decreased Ankle Osteoarthritis Scale (AOS) pain and disability subscales and high patient satisfaction after 24 months and 10 year follow-up.^77^ Interestingly, two studies both reported that the AOFAS score increased greatly in the first 24 months post-operatively, but after 48 months the AOFAS score was slightly decreased, yet remained significantly higher than the preoperative score.^77^^,^^78^ The reason behind this still needs further investigation.

## 5 Conclusion

In conclusion, there are several conservative and surgical approaches currently used to treat OLTs. All of these procedures have pros- and cons- and a decision has to be made based upon the likely efficacy compared with the potential risks e.g. donor site morbidity or risks associated with multiple surgical procedures. The judgement of which approach is most suited to each patient depends on the specific presentation of the defect e.g. size, site (contained or uncontained) depth and cyst presence. Moreover, for certain demographics of patient there will be greater impetus on choosing an approach which will allow for rapid return to normal physical activity, such as athletes. There remains a need to better predict which procedure is most likely to benefit an individual, perhaps through the use of imaging or fluid biomarkers, to ensure that repair of OLTs can be achieved as quickly as possible preventing further damage to the joint and the likelihood of developing OA.

## Funding

The authors would like to acknowledge 10.13039/501100012041Versus Arthritis (Grants 18480, 19429, 21156 and 20815), the 10.13039/501100000265Medical Research Council (MR/L010453/1, MR/N02706X/1 and MR/S015167/1) and the 10.13039/100015310Orthopaedic Institute Ltd (RPG 184) for supporting the salaries of the authors (TL, HM, CH and KW) and associated research.

## Declarations of competing interest

None.
